# The relation of general socio-emotional processing to parenting specific behavior: a study of mothers with and without posttraumatic stress disorder

**DOI:** 10.3389/fpsyg.2015.01575

**Published:** 2015-10-29

**Authors:** Dominik A. Moser, Tatjana Aue, Francesca Suardi, Aurélia Manini, Ana Sancho Rossignol, Maria I. Cordero, Gaëlle Merminod, François Ansermet, Sandra Rusconi Serpa, Nicolas Favez, Daniel S. Schechter

**Affiliations:** ^1^Faculty of Psychology and Education Sciences, University of GenevaGeneva, Switzerland; ^2^Research Unit, Department of Child and Adolescent Psychiatry, University of Geneva HospitalsGeneva, Switzerland; ^3^Institute of Psychology, University of BernBern, Switzerland; ^4^Faculty of Health, Psychology and Social Care, Manchester Metropolitan UniversityManchester, UK; ^5^Faculty of Medicine, University of GenevaGeneva, Switzerland

**Keywords:** fMRI, PTSD, parenting, socio-emotional information processing, maternal sensitivity

## Abstract

Socio-emotional information processing during everyday human interactions has been assumed to translate to social-emotional information processing when parenting a child. Yet, few studies have examined whether this is indeed the case. This study aimed to improve on this by connecting the functional neuroimaging data when seeing socio-emotional interactions that are not parenting specific to observed maternal sensitivity. The current study considered 45 mothers of small children (12–42 months of age). It included healthy controls (HC) and mothers with interpersonal violence-related posttraumatic stress disorder (IPV-PTSD), as well as mothers without PTSD, both with and without IPV exposure. We found that anterior cingulate cortex (ACC) and ventromedial prefrontal cortex (vmPFC) activity correlated negatively with observed maternal sensitivity when mothers watched videos of menacing vs. prosocial adult male–female interactions. This relationship was independent of whether mothers were HC or had IPV-PTSD. We also found dorsolateral prefrontal cortex (dlPFC) activity to be correlated negatively with maternal sensitivity when mothers watched any kind of arousing adult interactions. With regards to ACC and vmPFC activity, we interpret our results to mean that the ease of general emotional information integration translates to parenting-specific behavior. Our dlPFC activity findings support the idea that the efficiency of top-down control of socio-emotional processing in non-parenting specific contexts may be predictive of parenting behavior.

## Introduction

Despite multiple clinical observations supporting that a mother's capacity to perceive and regulate emotion is crucial for sensitive parenting, there is surprisingly a dearth of empirical research confirming those observations (Newman et al., 2007; Arteche et al., 2011; Schechter et al., 2015). The latter is true despite the fact that several studies have examined how parents' cognitive and socio-emotional capacities predict psychological outcome variables such as impulse control, hostile aggression and attachment security and organization in their children (Barrett and Fleming, 2011; Goodman et al., 2011; Lyons-Ruth et al., 2013; Mazursky-Horowitz et al., 2015).

The following question thus remains to be answered empirically: How might parents' ways of processing socio-emotional information during daily interactions, what we shall refer to as “general socio-emotional information processing” (GSEIP, i.e., emotion perception, appraisal, reappraisal etc.), translate to socio-emotional information processing and behavior that is specific to the parent-child relationship. Parenting-specific behavior can be seen as an index of parenting specific processing and we thus refer to it as “parenting specific socio-emotional processing and behavior” (PSEIPB). General psychological factors/constructs such as emotion regulation, other executive functions or empathy may or may not influence parental behavior. While it seems likely that such factors underlie PSEIPB, this deserves further testing.

Because of the abundance of studies linking parental pathology to parental behavior and of studies linking parenting related processing to emotional capacities in the parenting context, many professionals in the field may have underestimated the importance of the link between GSEIP and PSEIPB. This underestimation is possibly the cause that very few studies to our knowledge intentionally performed tests of the association between GSEIP and actual parenting behavior by including both kinds of measures. While there is a rich literature that has looked at maternal sensitivity and infant outcome GSEIP (Feldman et al., 2004, 2011; Blair et al., 2006; Braungart-Rieker et al., 2010; Conradt and Ablow, 2010; Hirschler-Guttenberg et al., 2015), there is less literature linking maternal sensitivity to maternal emotion regulation and executive functions in a exclusively parenting-specific context (Borelli et al., 2012; Stacks et al., 2014; Schechter et al., 2015). Moreover, there is almost no literature concerned with linking maternal sensitivity to GSEIP outside of a parenting-specific context.

Better understanding of the link between GSEIP and PSEIPB would help to explain why some parents develop disturbances in their relationship with their children that compromise the latter's development of self-regulation of emotion and arousal, and thus would help in the development of preventive interventions that could facilitate parental participation in the mutual regulation of emotion and arousal with their infants and young children. To do so, it is important to take into account the relationship of GSEIP not only on parental processing in the brain (processes related to preoccupations that are due to having and taking care of children) but more proximally on parental behavior.

No study to our knowledge has so far tested the link between a non-parenting related socio-emotional information task via the use of neuroimaging and actual parenting behavior. Neuroimaging is one option for the generation of hypotheses as to whether GSEIP translates to PSEIPB because it allows us to investigate whether brain regions that are linked to a task that is not specific to parenting are implicated in PSEIPB. We developed our to test the hypothesis that brain-processing of the information provided in a socio-emotional interaction among adult men and women is significantly associated with maternal sensitivity when mothers play with their children.

### PSEIPB and neuroimaging

Most imaging studies on parenting have focused on stimuli that are closely linked to PSEIPB, using either auditory child signals (such as baby cries) (Lorberbaum et al., 2002; Kim et al., 2011; Laurent and Ablow, 2012; De Pisapia et al., 2013) or visual child signals such as facial expressions or behavior (Noriuchi et al., 2008; Strathearn et al., 2009; Mascaro et al., 2013; Moser et al., 2013; Swain et al., 2014). Imaging research on parenting has provided evidence that parenting relies on brain regions that are also important for the integration of emotional information and emotion regulation functions. One study looked at how neural activation related to maternal behavior (Atzil et al., 2011). It revealed that the parenting style displayed was linked to the connectivity between the limbic system and the medial prefrontal cortex (mPFC) at the viewing of one's own child. Synchronous mothers, namely mothers who correctly modulated their behavior in accordance with child needs and signals, displayed a functional connection between the mPFC and the left nucleus accumbens. Intrusive mothers, namely mothers who tended to disregard child signals, rather demonstrated a functional connection between the mPFC and the right amygdala. The authors of that study interpreted these results as evidence that regions that are involved both in emotion modulation and in motivational aspects of reward and stress, play an important role in parenting.

A recent study conducted by Ho et al. (2014), comes closest to connecting GSEIP to PSEIPB in neuroimaging. While this study did not link a GSEIP task in the scanner to observable parenting outside the scanner, it did associate a generally applicable GSEIP measure with a PSEIPB task in the scanner. Ho et al. (2014) linked dispositional empathy (i.e., as a measure of GSEIP) prior to fMRI to a parental decision-making task involving negative and positive feedback toward children performed in an MRI scanner, within a sample of 14 mothers. Neural activity in the supplementary motor area, amygdala, and ventrolateral prefrontal cortex during the parental decision-making task was linked to different empathic dispositions. This suggested that differing empathic dispositions may change the way feedback toward children is processed.

Psychopathology that affects GSEIP has also been looked at in the context of parenting and neuroimaging. Mothers with greater anxiety and more negative mood showed less amygdala response while seeing their children in emotionally positive situations. This diminished amygdala response was also correlated with more negatively valenced parenting attitudes and experiences (Barrett et al., 2012). With the amygdala's important role in signaling saliency, this possibly suggests that mothers who are less anxious and stressed about being a parent perceive the positive emotions of their child as more salient. Furthermore, greater depressive symptoms have been associated with diminished activity in the orbitofrontal cortex (OFC) and dorsal anterior cingulate cortex (ACC) as well as the superior frontal gyrus, when mothers hear their own vs. other babies' cry (Laurent and Ablow, 2012). These regions have been implicated in the information processing of both emotional valence and salience (Etkin et al., 2011). Mothers with interpersonal violence-related post-traumatic stress disorder (IPV-PTSD), a form of psychopathology that diversely impacts emotion regulation, may feel more stressed when observing children in distress. IPV-PTSD mothers showed increased activation in the anterior entorhinal cortex when seeing children during separation compared to play, with less top-down regulation within the brain by dorsal prefrontal regions (Schechter et al., 2012), suggesting altered control of fear and emotion circuits by regions involved in executive control. In sum, the literature on mothering and brain imaging suggests an important role of regions particularly involved in emotional saliency and valence processing as well as emotion regulation. There are studies that have shown that a link between psychopathologies related to GSEIP neural activity in the PSEIPB related context exist (Moses-Kolko et al., 2010; Ho et al., 2014). To our knowledge, however, there have been no studies looking at the reverse approach—whether the neural activation in tasks not directly related to parenting is associated with actual parenting behavior outside of the scanner, neither in healthy controls (HC), nor in pathological participants.

### The current study

In the present study we aimed to test the hypothesis that there are observable patterns of brain activity which, when correlated to rater-assessed (i.e., objective) maternal sensitivity, support generalization from GSEIP to PSEIPB, irrespective of whether or not mothers have diagnosed psychopathology. This was based on the assumption that the same basic processes at work in GSEIP are also necessary for maternal sensitivity, as in Ainsworth's original description (Ainsworth and Witing, 1969) of maternal sensitivity. Additionally we examined the impact of interpersonal violence-related post-traumatic stress disorder (IPV-PTSD) and whether or not potential links between GSEIP and PSEIPB were independent from or altered in IPV-PTSD. IPV-PTSD particularly interested us as form of psychopathology, as women with IPV-PTSD can be expected to activate a fear-conditioned response and thus alter information processing when they see menacing male–female interactions, such as are shown in our GSEIP task (Moser et al., 2015)^1^.

We believe that this study would potentially advance research in the field in three important ways: (1) It will examine the relationship socio-emotional interaction stimuli that are not related to parenting and parenting itself. (2) It will examine whether impaired PSEIPB in mothers with IPV-PTSD are specific to the IPV-PTSD population, or whether they are just more severe forms of an association that exists in a similar, but less disadvantageous, form in the general population. (3) For HC there are—as far as we know- no studies relating emotion tasks that are independent of participants' role as mothers to maternal sensitivity that was acquired by independent observers (instead of questionnaires). This study will thus significantly enlarge our knowledge about how emotion-related parental brain activity is related to parenting capacities themselves.

We hypothesized that, even when accounting for IPV-PTSD symptom severity, neural activity in response to our task depicting emotional scenes of adult male–female interaction would be associated with maternal sensitivity as an index for PSEIPB—this, in spite of the fact that our task was not directly child-related but rather related to GSEIP. Our hypothesis would thus be in accordance with the generally held assumption that GSEIP capacities translate to PSEIPB, no matter whether there is psychopathology or not. We predict that such an association between maternal sensitivity and neural activity could be found in regions that have previously been implicated both in GSEIP and in parenting. These regions specifically include the mPFC, the ACC, the amygdala, the OFC, and the insula (Swain et al., 2014).

## Methods

### Recruitment

Participants were recruited via flyers posted within buildings of the hospitals and the University of Geneva as well as within community centers, daycare places, schools, domestic violence agencies, and shelters. Participants gave informed consent before participation in the study. This MRI study was nested within an ongoing study of interpersonal violence and intergenerational transmission of related trauma and psychopathology (Schechter and Rusconi, 2014) and approved by the Institutional Review Board of the University of Geneva Hospitals and Faculty of Medicine. Forty-eight mothers of young children (12–42 months of age) who were eligible for an MRI scan participated. Two participants were excluded from analysis due to excessive motion in the scanner. One participant was excluded due to problems with her behavioral data (contradictory answers at different time points).

### Instruments

Maternal sensitivity (i.e., PSEIPB) was measured via structured behavioral observations during 5 min of videotaped mother-child play which were taped during the pre-MRI procedure. Two raters (blind to group) who were psychologists and trained to reliability on the CARE-Index (Crittenden, unpublished manuscript) coded maternal behavior. Inter-rater reliability was very high (*k* = 0.86).

During an initial videotaped interview, that was part of the pre-MRI protocol participants underwent a variety of psychometrics including the Clinician Administered PTSD Scale (CAPS) (Blake et al., 1995) to assess life time PTSD and the Posttraumatic Symptom Checklist—short version (PCL-S) to assess current PTSD symptoms. Participants were diagnosed as having IPV-PTSD if their CAPS score was 55 and above and their PCL-S score was 40 and above; and as HC if their CAPS score was below 30, and their PCL-S score was below 25. If one of their scores was in between those values they were classified as sub-threshold IPV-PTSD.

### Group characteristics

Of the analyzed participants, 17 were diagnosed with IPV-PTSD (mean age = 32.8; SD = 5.7), 21 were HC (mean age = 34.7; SD = 5), and seven mothers did not belong to either group (i.e., had subthreshold IPV-PTSD; 32.8 SD = 4.7). Information on the criteria of diagnosis can be found in the Supplementary Materials. Groups did not differ significantly from each other with respect to age [*F*_(2, 42)_ = 0.221, *p* = 0.803]. All participants classified as IPV-PTSD and subthreshold had experienced traumatic life events (i.e., violence) as adults, while 33% of HC had experienced at least one type of physical or sexual form of violence. As expected, groups differed significantly in terms of maternal sensitivity [*F*_(2, 42)_ = 10.457, *p* < 0.01], primarily due to IPV-PTSD mothers (mean = 4.53; SD = 1.12) having lower sensitivity than subthresholds (mean = 5.71; SD = 1.38) and HC (mean = 6.19; SD = 1.03). Compared to HC (mean = 4.33; SD = 2.00), IPV-PTSD participants (mean = 5.88, SD = 2.01) had a significantly lower SES [high scores indicate low status; *t*_(36)_ = −2.34, *p* = 0.025]. SES was assessed with the Geneva Sociodemographic Questionnaire (Sancho Rossignol et al., 2010), which assesses level of education and professional status.

### Procedure

#### Procedures for visits prior to MRI scan

Participants received documentation that detailed the study procedure and gave thereafter informed consent. This was in accordance with the Helsinki Declaration of Human Rights (World Medical Association, 1999). The pre-MRI protocol is further described in the Supplemental Materials.

#### MRI-procedure (i.e., GSEIP)

Participants saw 23 silent movies of 20 s duration each in pseudo-randomized order. All movie excerpts showed a male and a female interacting with each other. These excerpts were chosen and grouped into 3 conditions based on the results of a cluster analysis of a validation study described in Moser et al. (2015): eight excerpts had been rated as displaying menace and strongly negative affect, eight displayed prosocial interaction and moderately to highly positive affect, and seven displayed neutral affect rated to display low arousal and neutral valence. Neutral scenes depicted an adult male and female in conversation or jointly attending to an event with neither an excess of positive affect, romantic content, nor negative affect and threat. After each movie, participants were asked to rate the valence of the predominant emotion that the movie elicited on a scale of 1 (very negative) to 7 (very positive) and then to rate the arousal level of that emotion again on a scale of 1 (no arousal) to 7 (maximum arousal). The time-limit for those judgments was 4 s. Participants practiced the rating procedure prior to entering the MRI scanner. Further information concerning the MRI data acquisition and preprocessing can be found in the Supplemental Materials.

### Data analyses

Imaging analysis at the first level was focused on the average brain activity of three main conditions: menacing, prosocial, and neutral male–female interactions. A first level t-map for menacing compared with prosocial scenes was initially performed for each subject in order to test for the influence of negative and positive valence. The same was done for a second contrast of interest: emotional vs. neutral, with emotional scenes combining menacing and prosocial scenes. Brain activation during the arousal and valence judgments of the movies was modeled but not analyzed.

The first level whole-brain contrasts were then used to assess how well maternal sensitivity—independently of IPV-PTSD—was predicted from brain activation in the GSEIP paradigm. Two regressions including all participants were performed for each voxel. They used the contrasts of neural activity as the dependent and maternal sensitivity as an independent variable, with IPV-PTSD symptom severity as a covariate. IPV-PTSD symptom severity was defined as the mean value of the PCL-S and CAPS after they had been z-standardized. In order to have a better idea whether effects originated from IPV-PTSD and/or HC, we performed *post-hoc* tests, calculating the same correlation separately within those two groups, for every cluster for which we had found significant effects. We left out a group-specific analysis of the subthreshold sample due to the small number of participants therein.

For our whole-brain analysis the threshold of significance was defined as an uncorrected *p* < 0.005 with cluster-sizes of at least 27 contiguous voxels (for details see Supplemental Materials).

Since there was a significant group difference in SES (i.e., IPV-PTSD < HC), we initially entered SES as a covariate in all our statistical analyses. Because SES did not significantly alter the model, SES is not included in any reported analysis.

Finally, for each cluster we extracted average activity values to SPSS and calculated a Pearson product moment correlation coefficient regarding the association of the BOLD effect with these ratings of arousal and valence. We did this for the whole group, but also for both HC and IPV-PTSD groups separately (Supplemental Tables 1–3).

In order to control for the possibility that maternal sensitivity could be predicted by individual differences in subjective arousal or valence, we additionally calculated Pearson product moment correlation coefficients between maternal sensitivity and the evaluation of the arousal and valence levels of each movie condition (menacing, neutral, and prosocial) as well as the differences between these movie conditions analogous to the MRI data (menacing vs. prosocial and emotional vs. neutral). This analysis was performed for the whole group, but also for both HC and IPV-PTSD separately.

## Results

### Menacing vs. prosocial contrast

**Figure 1:**
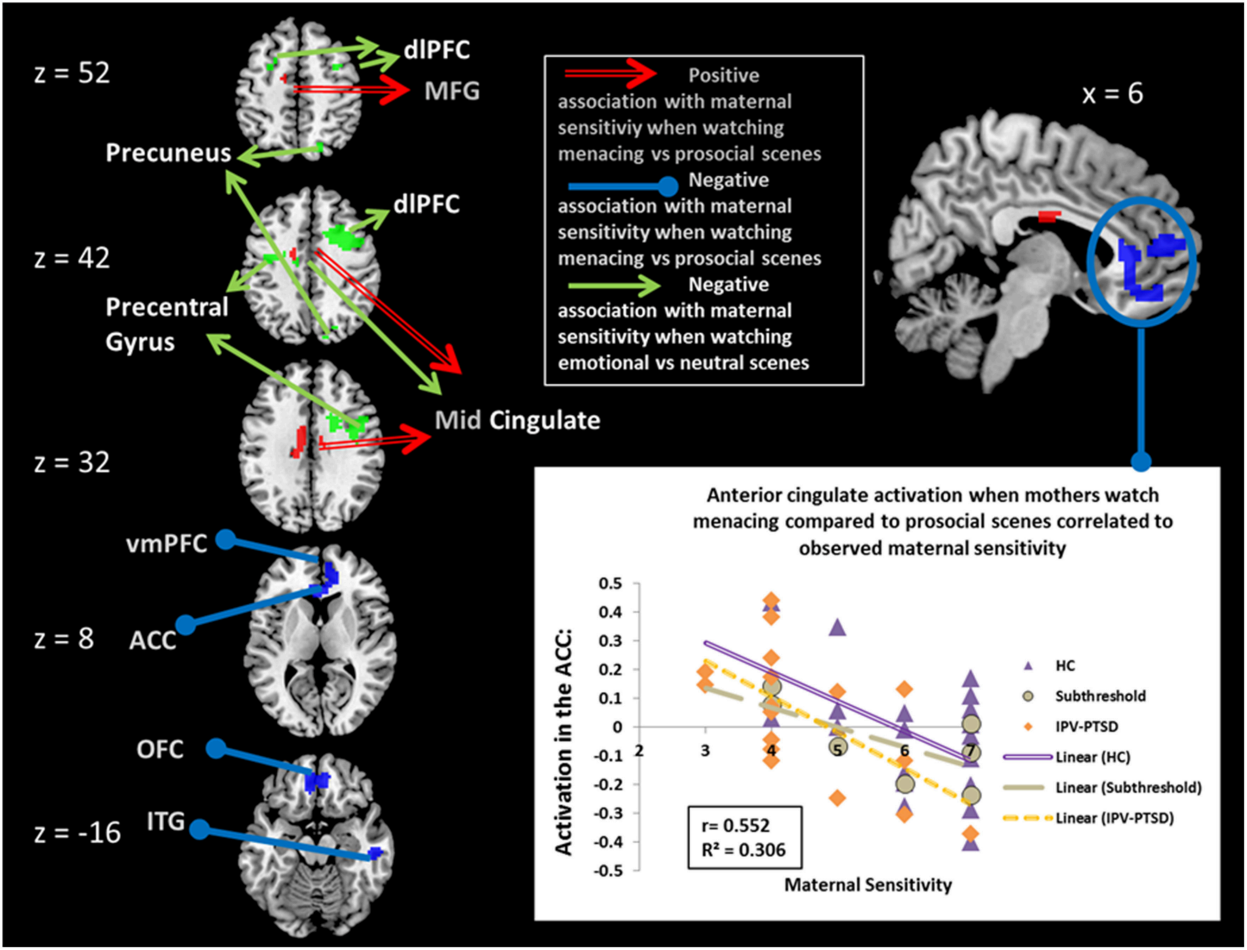
**Partial correlations of maternal sensitivity with BOLD effects corrected for IPV-PTSD symptom severity**. Red, positive association when mothers watch scenes of menacing vs. prosocial male–female interactions; blue, negative association when mothers watch scenes of menacing vs. prosocial male–female interaction; green, negative association when mothers watch scenes of emotional (menacing and prosocial scenes combined) vs. neutral male–female interaction. dlPFC, dorsolateral Prefrontal Cortex; MFG, Medial Frontal Gyrus; vmPFC, ventromedial Prefrontal Cortex; ACC, Anterior Cingulate Cortex; OFC, Orbitofrontal Cortex; ITG, Inferior Temporal Gyrus.

When correcting for IPV-PTSD symptom severity, maternal sensitivity was negatively associated with brain activation in a cluster in the right inferior temporal gyrus and in another cluster that comprised parts of the ventral ACC (vACC; Figure 1), ventromedial prefrontal cortex (vmPFC), and medial OFC. *Post-hoc* analyses suggested that the effect within this latter cluster was driven by significant correlations for both the IPV-PTSD group and the control group, and that primarily the prosocial (positive partial correlation) and to a smaller degree also the menacing condition (negative partial correlation) had contributed to the effect. Within this vACC/vmPFC cluster, only the activity during menacing but not that during prosocial scenes was significantly different from that of neutral scenes. Activity in this cluster in the vACC/vmPFC was not significantly correlated with subjective valence or arousal (see Supplemental Table 1).

When correcting for maternal IPV-PTSD symptom severity, maternal sensitivity was positively associated with neural activity in response to menacing compared to prosocial scenes in the middle cingulate cortex, the left inferior frontal gyrus, and a cluster comprising the most posterior part of the left medial frontal gyrus and the mid-cingulate cortex. *Post-hoc* analyses showed that all of these latter clusters were only significant within HC (see Table 1).

**Table 1:** **Significant correlations, between maternal sensitivity and BOLD activations when mothers see scenes of menacing vs. prosocial adult male–female interactions, corrected IPV-PTSD symptom severity**.

**Cluster size**	**Peak voxel *t***	**Peak voxel *p***	**MNI coordinates**			**Regions comprised in this cluster**	**Partial correlations for the overall cluster**		
			**x**	**y**	**z**		**For the whole sample**	***Post-hoc*** **tests**	
								**Within IPV-PTSD**	**Within HC**
**POSITIVE ASSOCIATIONS OF BOLD ACTIVITY WITH MATERNAL SENSITIVITY**									
92	3.49	0.001	−9	−1	28	Mid cingulate	*r* = 0.471, *p* = 0.001	*r* = 0.324, *p* = 0.221	*r* = 0.501, *p* = 0.024
27	3.60	<0.001	−48	17	1	Left IFG	*r* = 0.461, *p* = 0.002	*r* = 0.141, *p* = 0.603	*r* = 0.551, *p* = 0.012
31	3.31	0.001	−15	−7	52	MFG/Mid cingulate	*r* = 0.467, *p* = 0.001	*r* = 0.368, *p* = 0.161	*r* = 0.447, *p* = 0.048
**NEGATIVE ASSOCIATIONS OF BOLD ACTIVITY WITH MATERNAL SENSITIVITY**									
411	4.09	<0.001	−6	29	10	vACC, vmPFC, mOFC	*r* = −0.557, *p* < 0.001	*r* = −0.613, *p* = 0.012	*r* = −0.536, *p* = 0.015
41	4.15	<0.001	54	−22	−20	Right inferior temporal gyrus	*r* = −0.508, *p* < 0.001	*r* = −0.639, *p* = 0.008	*r* = −0.495, *p* = 0.027

*IFG, Inferior Frontal Gyrus; HC, healthy controls; IPV-PTSD, interpersonal violence related post traumatic stress disorder; MFG, Medial Frontal Gyrus; mOFC, medial Orbitofrontal Cortex; vACC, ventral anterior Cingulate; vmPFC, dorsolateral Prefrontal Cortex*.

### Emotional vs. neutral contrast

In response to emotional compared to neutral scenes, there were no significant positive associations between neural activation and maternal sensitivity. Negative associations could be found in clusters in the mid-cingulate cortex, the bilateral dorsolateral prefrontal cortex (dlPFC), and the precentral gyrus (see Table 2 and Supplemental Table 5). These effects were driven primarily by the HC group, and both the menacing and the prosocial conditions contributed; yet, only menacing conditions led to significantly more activation than neutral ones, while prosocial ones did not (see Table 2). *Post-hoc* analyses revealed that, within IPV-PTSD but not within HC, there were negative correlations between subjective arousal for menacing vs. prosocial scenes and brain activation in one cluster in the left dlPFC (*n* = 17, *r* = −0.638, *p* = 0.006) and the cluster containing the right dlPFC (*n* = 17, *r* = −0.511, *p* = 0.036, see also Supplemental Table 3).

**Table 2:** **Significant correlations, between maternal sensitivity and BOLD activations when mothers see scenes of emotional (menacing and prosocial combined) vs. neutral adult male–female interactions, corrected IPV-PTSD symptom severity**.

**Cluster size**	**Peak voxel *t***	**Peak voxel *p***	**MNI coordinates**			**Regions comprised in this cluster**	**Partial correlations for the overall cluster**		
			**x**	**y**	**z**		**For the whole sample**	***Post-hoc*** **tests**	
								**Within IPV-PTSD**	**Within HC**
**NEGATIVE ASSOCIATIONS OF BOLD ACTIVITY WITH MATERNAL SENSITIVITY**									
496	4.23	<0.001	42	2	28	Right PrcG, Mid cingulate, Right dlPFC	*r* = −0.556, *p* < 0.001	*r* = −0.339, *p* = 0.199	*r* = −0.620, *p* = 0.004
32	3.29	0.001	−45	8	25	Left dlPFC	*r* = −0.421, *p* = 0.004	*r* = −0.164, *p* = 0.543	*r* = −0.566, *p* = 0.009
27	3.19	0.001	−27	5	58	Left dlPFC	*r* = −0.437, *p* = 0.003	*r* = −0.160, *p* = 0.544	*r* = −0.491, *p* = 0.028
41	3.25	0.001	−39	−10	43	Left PrcG, Mid cingulate	*r* = −0.464, *p* = 0.002	*r* = −0.319, *p* = 0.229	*r* = −0.560, *p* = 0.010
35	3.17	0.001	18	−70	52	Right PrcG	*r* = −0.463, *p* = 0.002	*r* = −0.021 *p* = −0.540	*r* = −0.540, *p* = 0.014

*dlPFC, dorsolateral Prefrontal Cortex; HC, healthy controls; IPV-PTSD, interpersonal violence related post traumatic stress disorder; PrcG, Precentral Gyrus*.

Maternal sensitivity did not correlate with valence or arousal judgments of the differing movie conditions or their contrasts (for the group as a whole: all ps > 0.218, for the HC: all ps > 0.077, for IPV-PTSD; all ps > 0.414).

## Discussion

### Menacing vs. prosocial contrast

Our study successfully linked PSEIPB, as measured by maternal sensitivity, to decreased activity in the vACC, vmPFC and mOFC in GSEIP, as measured when mothers watched adult male–female interaction that portrayed menacing vs. prosocial interactions. *Post-hoc* tests suggested that both the menacing and prosocial conditions had contributed to this finding, but that for vACC/vmPFC/ mOFC activity, only the menacing condition was significantly different from neutral activity. Ventral-rostral portions of the ACC and mPFC have a regulatory function in relation to the limbic regions that are involved in generating emotional responses (Etkin et al., 2011; Motzkin et al., 2015). Moreover, vmPFC and OFC have been related to the encoding of emotional value as it is affected by conscious emotion regulation strategies both for positive (Winecoff et al., 2013) and negative affect (Diekhof et al., 2011), indicating that it is not just the simple emotional value of the stimulus, that influences the activity of the vmPFC and OFC, but rather the value of the stimulus in a specific context to a specific person Along these lines, a recent study suggested that backgrounds containing emotional value (such as a house on fire or a holiday cottage) interact strongly with emotion-specific face processing upon the activation of the subgenual ACC (Van den Stock et al., 2014). Of note, in a meta-analysis, OFC activation has been shown to have a stronger relation to complex emotional scenes even more than to stimuli showing exclusively emotional faces (Sabatinelli et al., 2011). This indicates a role of the OFC particularly in the face of complex stimuli that include naturalistic scenes and emotional non-verbal communication via gesture, bodily movement, tone and rhythmicity, such as ours.

In sum, these regions are central to the integration of socio-emotional information, and the emotion regulation in response to complex emotional information, and thus are crucial for GSEIP. We speculate that the fact that higher activation in the ACC during menacing vs. prosocial scenes is associated with decreasing maternal sensitivity, indicates that GSEIP of menacing scenes relies on more effortful processing in mothers who are less sensitive than in mothers who are more sensitive. Toddlers often express and act upon their negative emotions without the capacity to inhibit or modulate their communication, and thus make particular demands on maternal emotion regulation. This leads us to the interpretation that if emotion processing of aversive emotions excessively strains processing resources, it will be difficult to find additional processing resources necessary to both regulate the mother's own emotions and to perceive and react sensitively to the child's emotions (i.e., when confronted with a toddler in distress). This notion has been supported by clinical research observations in which traumatized mothers were less available for joint attention with their children following separation stress than during free-play prior to separation (Schechter et al., 2010).

Alternatively one could speculate that this result is due to HC mothers who may be better able to evaluate (and enjoy) the prosocial interactions, and this too may translate to maternal sensitivity, potentially due to a better use of available resources. In this sense mothers with IPV-PTSD may be at a disadvantage here, since in their particular experience even prosocial experiences with men were often preludes to violence.

Interestingly the correlation of ACC activation and maternal sensitivity was not specific to women with either IPV-PTSD or HC. This suggests that while ACC activation in IPV-PTSD generally differentiates from HC (Moser et al., 2015), it does not constitute an HC or IPV-PTSD-specific activation that differentiates sensitive mothers from less sensitive ones. However, because IPV-PTSD itself is generally associated with decreased ACC activation when mothers see arousing scenes (Moser et al., 2015), IPV-PTSD served as a good predictor for maternal sensitivity for the group as a whole. This observation can be related to a finding in a previous study that suggested that adverse early life events do not have a direct effect on maternal sensitivity, but do have an effect if modulated via hypothalamic pituitary-adrenal function (Gonzalez et al., 2012). These findings together suggest that adverse life events seem to impact PSEIPB only if the accompanying problems also affect the organism enough to change its stress-related psychobiology. While several studies have explored this issue (Schechter et al., 2008; Sturge-Apple et al., 2010; Parlar et al., 2014), more specific research to test this hypothesis is needed.

We found that maternal sensitivity was positively related to neural activity in the mid-cingulate cortex when watching menacing compared to prosocial scenes, and that it was negatively related to neural activity in the mid-cingulate, when watching either menacing or prosocial emotional stimuli compared to neutral stimuli. Less sensitive mothers increased mid-cingulate activity during prosocial scenes, rather than decreased it in response to menacing scenes. We interpreted this to mean that within the mid-cingulate, maternal neural response to negative valence and general arousal do not predict maternal sensitivity. Rather, the processing of positive emotion may be more salient for sensitive mothers.

The literature on empathy has consistently found the mid-cingulate to be activated in empathy paradigms (Bernhardt and Singer, 2012). Mid-cingulate activation has also been found to correlate positively with subjectively felt pain intensity (Kong et al., 2008). Empathy toward children is a clearly integral to sensitive parenting (Emery et al., 2014).

### Emotional vs. neutral contrast

We found that bilateral dlPFC activation when seeing any emotional scene was negatively associated with observed maternal sensitivity. The dlPFC is a region implicated in executive functions that among other functions involve conscious emotional control (Buhle et al., 2014), but has also been connected to reappraisal of arousing emotions (Silvers et al., 2015). The effect was primarily driven by HC. Either less need for strategies to alter GSEIP such as reappraisal or more efficient use of such strategies may thus be adaptive and supportive of PSEIPB in healthy individuals (HC). Since we could not find a correlation between arousal or valence levels reported by HC and neural activation in the dlPFC, we tend to favor the latter hypothesis. Within our data, this relationship between GSEIP activation in the dlPFC and PSEIPB does not hold for the IPV-PTSD group. While it is possible that this is due to a lack of statistical power, a previous study found that IPV-PTSD was associated with changes in activation in the dorsal PFC regions, as a function of the valence, but not arousal-level, of the stimuli (Moser et al., 2015). This suggests that when women are affected by IPV-PTSD, the dlPFCs altered role in GSEIP emotion regulation may also change the relationship between GSEIP and maternal sensitivity.

## Conclusions

Overall, our study suggests that general socio-emotional information processing (“GSEIP”) in response to viewing adult male–female interaction stimuli during fMRI translates to caregiving behavior during laboratory observations of mother-child play interactions. Neural activity within the ACC and vmPFC in response to film-scenes of menacing vs. prosocial adult male–female interactions correlated negatively with maternal sensitivity during play interactions with her child. The latter is consistent with the idea that mothers' integration of complex emotional information in response to socio-emotional information processing of adult-interactions translated to their emotion perception, appraisal, interpretation, and behavioral response with their children. The basic mechanism responsible for this translation of GSEIP to PSEIPB was similar in HC and IPV-PTSD. But as a previous study of ours revealed (Moser et al., 2015), women suffering from IPV-PTSD generally show diminished activity of the ACC/vmPFC when reacting to arousing scenes. The present study suggests that this altered pattern of ACC/vmPFC activity may put mothers with IPV-PTSD at a disadvantage with respect to their capacity to self-regulate their emotions and thus to be able to engage in mutual emotion regulation with their children and to remain sensitive to their children's emotional communication.

Our finding that a pattern of dlPFC activation related to maternal sensitivity, suggests that top-down regulation GSEIP translates in PSEIPB, at least among HC. We interpreted this finding to mean that HC mothers with higher maternal sensitivity were more efficient when applying conscious strategies in order to alter GSEIP that focused on perceived arousal. Future studies, might thus focus on whether intervention that is centered on improving GSEIP (i.e., perception and comprehension of emotional content) might further improve resources in HC, and be a useful addition to parent-child psychotherapy.

The results of this study shed light on how, what we have termed as “GSEIP” translates to “PSEIPB.” Although the implied direction of this translation seems reasonable to us, this is not proven here, since our results are correlational in nature. Concerning the causalities of the study's effects, bi-directionality between GSEIP and PSEIPB as well as a third factor causally mediating both GSEIP and PSEIPB remain theoretically viable possibilities. Yet, the translation from GSEIP to PSEIPB seems reasonable to us as it is in line with most of the literatures assumptions.

## Limitations

The current paper did not take into account whether the children differed with respect to their behavior. We cannot answer the question as to whether there is an interaction between child behavior, maternal behavior and maternal brain activation in this experiment. We also did not ask our participants about the emotion regulation strategies they employed while being in the scanner. As such, we do not know if differing degrees of conscious emotion regulation strategies may have played a role in our findings.

While the study did control for IPV-PTSD symptom severity and its impact on neural activity, it did not do so for violence exposure. While IPV-PTSD symptom severity is correlated to violence exposure such that worse and more chronic exposure correlates to higher symptoms and previous studies found IPV-PTSD symptoms to be more predictive than violence exposure (Schechter et al., 2005, 2007), we cannot fully exclude that violence exposure by itself would also have affected the results. This may also be true for controls, 33% of whom had experienced some sort of violence (usually singularly and not repeated) even though they did not report any clinically significant IPV-PTSD symptoms.

It is further possible that there remain other variables not controlled for in this study; variables that are common to both PSEIPB and GSEIP, and that are causal to associations between these two factors as described in our study. Future studies combining GSEIP and PSEIPB tasks, may also want to combine behavioral measures additionally to the fMRI measure on a GSEIP task, as well as an fMRI-PSEIPB task in addition to the observed parenting quality, in order to measure links more directly. Finally, the sample size for this study was primarily determined by the available participants. A bigger sample size with increased statistical power might have provided additional significant effects.

### Conflict of interest statement

The authors declare that the research was conducted in the absence of any commercial or financial relationships that could be construed as a potential conflict of interest.

## Footnotes

^1^In a previous analysis, we investigated a more general research question that focused on whether women with IPV-PTSD differed from controls when observing such interactions (Moser et al., 2015). An overlapping sample of that study is re-analyzed, but significantly extended here, by adding data on maternal sensitivity.
