# Supplementary material for: The relation of general socio-emotional processing to parenting specific behavior: a study of mothers with and without posttraumatic stress disorder
Source: Front Psychol. 2015 Oct 29;6:1575. doi: 10.3389/fpsyg.2015.01575 (PMC4625041; doi:10.3389/fpsyg.2015.01575)
Supplement: Supplementary file 1 [file Presentation1.PDF]

Supplemental Materials

Procedure

The pre-MRI protocol consisted of an hour-long screening plus two visits of two hours each. The first of those visits included structured and semi-structured clinical interviews. The second of those visits included a behavioral protocol with participant mothers and children. Mothers were asked to bring their child to the laboratory for a modified Crowell Procedure (Zeanah, 2000), which - among other things - included 8 minutes of free play for the dyad, followed by 3 minutes of separation, 2 minutes of reunion, 2 minutes of cleaning up, 4 minutes of structured play, and then again 3 minutes of separation before being followed by the presentation of novel toys and a stranger in a clown costume entering the room. This procedure was followed by administration of measures focusing on the child's life events, psychopathology, and socio-emotional development.

Image Acquisition and Pre-Processing

Scanning took place in with a scanner provided by the Geneva branch of the Center for Biomedical Imaging (CIBM) of the Geneva-Lausanne Universities, the EPFL and the Geneva-Lausanne University Hospitals. All images were acquired on a Siemens 3 Tesla TrioTim scanner using a 12 channel receiver. After a three plane localizing image, a T1-weighted, Spoiled Gradient Recall image was acquired in the sagittal plane to prescribe the location of the anterior commissure–posterior commissure (AC/PC) line. Axial functional images positioned parallel to the AC/PC line were obtained using a T2*-weighted gradient-recalled single-shot echo planar pulse sequence with TR=2100ms, TE=30ms, 80º flip angle, 25.6x25.6cm2 field of view, and a

64x64 voxel slice matrix. We acquired 36 slices of 3.5 mm thickness with a spacing of 0.5 mm to provide an effective resolution of 4 x 4 x 3.5 mm3. Slices were acquired in interleaved order and spanned the entire brain. The functional echo planar images were preprocessed and statistically analyzed using batch programming based on SPM8 under MATLABR2012a. Prior to analysis, images were visually inspected for major artifacts and signal dropout. Images were realigned to the middle slice of each scan. After motion correction, functional images were co-registered with the anatomical image of the same participant prior to being spatially normalized and reformatted into a 3x3x3 mm resolution space. Finally, normalized images were spatially smoothed using a Gaussian filter with a full-width half-maximum of 8mm.

Data Analyses

A Monte Carlo Simulation with 10,000 iterations indicated that the probability of finding an arbitrarily significant effect with an alpha of 0.05 is achieved when implementing the condition that a cluster of at least 27 contiguous voxels displays an effect of $p<.005$. Application of this threshold corrects for the multiple comparisons produced by the high number of voxels considered.

References

Zeanah, C., Larrieu JA, Heller SS, Vallier J. (2000). Infant-parent relationship assessment. In G. Press (Ed.), *Handboof of infant mental health* (pp. 222-235). New York.

Table 1

Correlations of mean BOLD activation in all clusters of Table 1 and 2 with judgments of arousal and valence within all participants.

Overall	Location Peak Voxel			Significance Stemming from	Arousal					Valence				
	X	Y	z		Scenes of Menace	Neutral Interactions	Prosocial Interactions	Menacing vs Prosocial Interactions	Emotional vs Neutral Interactions	Scenes of Menace	Neutral Interactions	Prosocial Interactions	Menacing vs Prosocial Interactions	Emotional vs Neutral Interactions
Mid Cingulate	-9	-1	28	Menace vs Prosocial	r = -0.097<br>p = 0.526	r = -0.027<br>p = 0.859	r = -0.003<br>p = 0.983	r = -0.095<br>p = 0.537	r = -0.044<br>p = 0.775	r = -0.044<br>p = 0.774	r = -0.018<br>p = 0.909	r = 0.088<br>p = 0.563	r = -0.082<br>p = 0.591	r = 0.036<br>p = 0.815
Left Inferior Frontal Gyrus	-48	17	1	Menace vs Prosocial	r = -0.007<br>p = 0.966	r = 0.115<br>p = 0.452	r = 0.125<br>p = 0.412	r = -0.109<br>p = 0.474	r = -0.070<br>p = 0.650	r = -0.020<br>p = 0.894	r = 0.075<br>p = 0.624	r = 0.108<br>p = 0.480	r = -0.078<br>p = 0.610	r = -0.076<br>p = 0.618
Medial Frontal Gyrus / Mid Cingulate	-15	-7	52	Menace vs Prosocial	r = -0.095<br>p = 0.534	r = 0.041<br>p = 0.788	r = 0.087<br>p = 0.568	r = -0.167<br>p = 0.272	r = -0.070<br>p = 0.646	r = 0.082<br>p = 0.592	r = -0.067<br>p = 0.664	r = 0.026<br>p = 0.868	r = 0.040<br>p = 0.796	r = -0.001<br>p = 0.993
Anterior Cingulate / vmPFC	-6	29	10	Prosocial vs Menace	r = 0.021<br>p = 0.892	r = 0.180<br>p = 0.236	r = 0.054<br>p = 0.724	r = -0.023<br>p = 0.878	r = -0.176<br>p = 0.247	r = 0.112<br>p = 0.462	r = 0.165<br>p = 0.277	r = -0.174<br>p = 0.253	r = 0.179<br>p = 0.239	r = -0.174<br>p = 0.253
Right Inferior Temporal Gyrus	54	-22	-20	Prosocial vs Menace	r = -0.012<br>p = 0.939	r = 0.124<br>p = 0.418	r = -0.050<br>p = 0.742	r = 0.030<br>p = 0.847	r = -0.197<br>p = 0.194	r = -0.028<br>p = 0.857	r = -0.097<br>p = 0.525	r = -0.158<br>p = 0.300	r = 0.076<br>p = 0.620	r = -0.166<br>p = 0.275
Mid Cingulate / Right dlPFC	42	2	28	Neutral vs Emotional	r = 0.178<br>p = 0.242	r = -0.005<br>p = 0.975	r = -0.066<br>p = 0.665	r = 0.233<br>p = 0.124	r = 0.103<br>p = 0.501	r = -0.121<br>p = 0.429	r = 0.044<br>p = 0.775	r = 0.059<br>p = 0.702	r = -0.116<br>p = 0.448	r = -0.019<br>p = 0.903
Left dlPFC	-45	8	25	Neutral vs Emotional	r = 0.132<br>p = 0.387	r = -0.078<br>p = 0.612	r = -0.062<br>p = 0.688	r = 0.183<br>p = 0.229	r = 0.162<br>p = 0.288	r = -0.273<br>p = 0.069	r = -0.038<br>p = 0.803	r = 0.011<br>p = 0.941	r = -0.189<br>p = 0.213	r = -0.019<br>p = 0.899
Left dlPFC	-27	5	58	Neutral vs Emotional	r = 0.181<br>p = 0.233	r = -0.151<br>p = 0.324	r = -0.154<br>p = 0.314	**r = 0.308**<br>**p = 0.040**	r = 0.233<br>p = 0.124	r = 0.005<br>p = 0.976	r = 0.017<br>p = 0.911	r = 0.097<br>p = 0.527	r = -0.055<br>p = 0.721	r = 0.164<br>p = 0.282
Left Precentral Gyrus / Mid Cingulate	-39	-10	43	Neutral vs Emotional	**r = 0.326**<br>**p = 0.029**	r = -0.021<br>p = 0.891	r = -0.041<br>p = 0.789	**r = 0.360**<br>**p = 0.015**	r = 0.255<br>p = 0.091	r = -0.238<br>p = 0.115	r = 0.011<br>p = 0.945	r = 0.111<br>p = 0.469	r = -0.225<br>p = 0.137	r = -0.028<br>p = 0.855
Right Precuneus	18	-70	52	Neutral vs Emotional	r = 0.025<br>p = 0.873	r = -0.044<br>p = 0.775	r = -0.105<br>p = 0.491	r = 0.111<br>p = 0.467	r = 0.007<br>p = 0.964	r = -0.097<br>p = 0.525	r = -0.121<br>p = 0.427	r = -0.144<br>p = 0.346	r = 0.021<br>p = 0.892	r = -0.037<br>p = 0.810

Significant correlations (uncorrected for multiple comparisons) are emphasized in bold type Abreviations: dlPFC = dorsolateral Prefrontal Cortex.

Table 2

Correlations of mean BOLD activation in all clusters of Table 1 and 2 with judgments of arousal and valence within the HC group.

HC	Location Peak Voxel			Significance Stemming from	Arousal					Valence				
	X	Y	z		Scenes of Menace	Neutral Interactions	Prosocial Interactions	Menacing vs Prosocial Interactions	Emotional vs Neutral Interactions	Scenes of Menace	Neutral Interactions	Prosocial Interactions	Menacing vs Prosocial Interactions	Emotional vs Neutral Interactions
Mid Cingulate	-9	-1	28	Menace vs Prosocial	r = -0.043<br>p = 0.854	r = -0.192<br>p = 0.406	r = -0.152<br>p = 0.511	r = 0.079<br>p = 0.734	r = 0.117<br>p = 0.613	r = 0.049<br>p = 0.832	r = -0.394<br>p = 0.077	r = -0.012<br>p = 0.957	r = -0.001<br>p = 0.996	r = 0.078<br>p = 0.736
Left Inferior Frontal Gyrus	-48	17	1	Menace vs Prosocial	r = 0.094<br>p = 0.686	r = -0.026<br>p = 0.912	r = -0.005<br>p = 0.981	r = 0.130<br>p = 0.576	r = 0.116<br>p = 0.616	r = 0.088<br>p = 0.706	r = 0.107<br>p = 0.644	r = 0.134<br>p = 0.564	r = 0.001<br>p = 0.996	r = 0.263<br>p = 0.249
Medial Frontal Gyrus / Mid Cingulate	-15	-7	52	Menace vs Prosocial	r = -0.102<br>p = 0.659	r = -0.059<br>p = 0.798	r = -0.025<br>p = 0.915	r = -0.114<br>p = 0.624	r = -0.032<br>p = 0.892	r = -0.004<br>p = 0.985	r = -0.241<br>p = 0.292	r = -0.009<br>p = 0.969	r = 0.120<br>p = 0.604	r = 0.235<br>p = 0.305
Anterior Cingulate / vmPFC	-6	29	10	Prosocial vs Menace	r = -0.027<br>p = 0.908	r = 0.280<br>p = 0.218	r = 0.072<br>p = 0.756	r = -0.100<br>p = 0.666	r = - 0.347<br>p = 0.124	r = 0.206<br>p = 0.370	r = 0.033<br>p = 0.887	r = 0.051<br>p = 0.825	r = - 0.372<br>p = 0.097	r = - 0.362<br>p = 0.107
Right Inferior Temporal Gyrus	54	-22	-20	Prosocial vs Menace	r = 0.219<br>p = 0.341	r = 0.224<br>p = 0.330	r = 0.163<br>p = 0.481	r = 0.145<br>p = 0.531	r = 0.009<br>p = 0.969	r = 0.179<br>p = 0.438	r = 0.249<br>p = 0.276	r = -0.372<br>p = 0.097	r = -0.102<br>p = 0.659	r = -0.276<br>p = 0.225
Mid Cingulate / Right dlPFC	42	2	28	Neutral vs Emotional	r = -0.079<br>p = 0.734	r = 0.079<br>p = 0.734	r = -0.012<br>p = 0.960	r = 0.094<br>p = 0.531	r = -0.183<br>p = 0.427	r = -0.070<br>p = 0.762	r = 0.253<br>p = 0.268	r = -0.039<br>p = 0.867	r = -0.115<br>p = 0.620	r = -0.261<br>p = 0.253
Left dlPFC	-45	8	25	Neutral vs Emotional	r = 0.012<br>p = 0.959	r = -0.220<br>p = 0.339	r = -0.222<br>p = 0.332	r = 0.215<br>p = 0.349	r = 0.161<br>p = 0.487	r = -0.162<br>p = 0.484	r = 0.150<br>p = 0.517	r = 0.056<br>p = 0.808	r = -0.139<br>p = 0.548	r = - 0.311<br>p = 0.170
Left dlPFC	-27	5	58	Neutral vs Emotional	r = -0.179<br>p = 0.437	r = -0.173<br>p = 0.452	r = -0.213<br>p = 0.355	r = -0.048<br>p = 0.836	r = -0.070<br>p = 0.764	r = -0.077<br>p = 0.739	r = 0.114<br>p = 0.621	r = 0.114<br>p = 0.623	r = 0.001<br>p = 0.997	r = 0.043<br>p = 0.852
Left Precentral Gyrus / Mid Cingulate	-39	-10	43	Neutral vs Emotional	r = -0.041<br>p = 0.861	r = 0.027<br>p = 0.909	r = -0.046<br>p = 0.843	r = -0.013<br>p = 0.956	r = -0.101<br>p = 0.665	r = -0.179<br>p = 0.437	r = 0.252<br>p = 0.270	r = 0.280<br>p = 0.219	r = -0.255<br>p = 0.264	r = -0.278<br>p = 0.222

Right Precuneus	18	-70	52	Neutral vs Emotional	r = -0.186 p = 0.419	r = -0.046 p = 0.844	r = -0.202 p = 0.379	r = -0.066 p = 0.776	r = -0.236 p = 0.302	r = -0.036 p = 0.875	r = 0.027 p = 0.907	r = -0.166 p = 0.471	r = -0.052 p = 0.823	r = -0.198 p = 0.390

Significant correlations (uncorrected for multiple comparisons) are emphasized in bold type Abreviations: dlPFC = dorsolateral Prefrontal Cortex.

Table 3

Correlations of mean BOLD activation in all clusters of Table 1 and 2 with judgments of arousal and valence within the IPV-PTSD group.

IPV-PTSD	Location Peak Voxel			Significance Stemming from	Arousal					Valence				
	X	Y	z		Scenes of Menace	Neutral Interactions	Prosocial Interactions	Menacing vs Prosocial Interactions	Emotional vs Neutral Interactions	Scenes of Menace	Neutral Interactions	Prosocial Interactions	Menacing vs Prosocial Interactions	Emotional vs Neutral Interactions
Mid Cingulate	-9	-1	28	Menace vs Prosocial	r = -0.101 p = 0.699	r = 0.130 p = 0.619	r = 0.050 p = 0.850	r = -0.120 p = 0.646	r = -0.173 p = 0.505	r = -0.349 p = 0.170	r = -0.227 p = 0.381	r = -0.066 p = 0.801	r = -0.177 p = 0.498	r = -0.60 p = 0.820
Left Inferior Frontal Gyrus	-48	17	1	Menace vs Prosocial	r = -0.199 p = 0.445	r = 0.265 p = 0.303	r = 0.128 p = 0.624	r = -0.258 p = 0.318	r = - 0.332 p = 0.193	r = 0.011 p = 0.968	r = 0.340 p = 0.181	r = 0.090 p = 0.732	r = -0.060 p = 0.819	r = -0.275 p = 0.286
Medial Frontal Gyrus / Mid Cingulate	-15	-7	52	Menace vs Prosocial	r = -0.255 p = 0.324	r = 0.353 p = 0.165	r = 0.426 p = 0.088	**r = -0.521 p = 0.032**	r = -0.281 p = 0.274	r = -0.168 p = 0.520	r = -0.018 p = 0.944	r = 0.104 p = 0.691	r = -0.186 p = 0.475	r = -0.014 p = 0.957
Anterior Cingulate / vmPFC	-6	29	10	Prosocial vs Menace	r = 0.133 p = 0.612	r = 0.070 p = 0.789	r = 0.046 p = 0.862	r = 0.077 p = 0.769	r = 0.046 p = 0.860	r = 0.018 p = 0.946	r = 0.150 p = 0.566	r = -0.077 p = 0.770	r = 0.069 p = 0.793	r = -0.205 p = 0.430
Right Inferior Temporal Gyrus	54	-22	-20	Prosocial vs Menace	r = -0.183 p = 0.482	r = 0.289 p = 0.261	r = -0.031 p = 0.906	r = -0.130 p = 0.620	r = -0.441 p = 0.076	r = 0.018 p = 0.946	r = 0.028 p = 0.915	r = -0.191 p = 0.463	r = 0.154 p = 0.555	r = -0.170 p = 0.515
Mid Cingulate / Right dlPFC	42	2	28	Neutral vs Emotional	**r = 0.555 p = 0.021**	r = -0.126 p = 0.631	r = -0.070 p = 0.789	**r = 0.511 p = 0.036**	r = 0.468 p = 0.058	r = -0.129 p = 0.623	r = 0.049 p = 0.852	r = 0.070 p = 0.789	r = -0.136 p = 0.604	r = -0.084 p = 0.748
Left dlPFC	-45	8	25	Neutral vs Emotional	r = 0.244 p = 0.346	r = 0.080 p = 0.760	r = 0.167 p = 0.523	r = 0.081 p = 0.757	r = 0.185 p = 0.477	r = -0.387 p = 0.125	r = -0.022 p = 0.934	r = 0.048 p = 0.855	r = -0.286 p = 0.265	r = -0.208 p = 0.422
Left dlPFC	-27	5	58	Neutral vs Emotional	**r = 0.630 p = 0.007**	r = -0.263 p = 0.307	r = -0.158 p = 0.545	**r = 0.638 p = 0.006**	**r = 0.608 p = 0.010**	r = 0.147 p = 0.573	r = 0.040 p = 0.878	r = 0.062 p = 0.814	r = 0.049 p = 0.851	r = 0.110 p = 0.675

Left Precentral Gyrus / Mid Cingulate	-39	-10	43	Neutral vs Emotional	**r = 0.733 p = 0.001**	r = -0.148 p = 0.570	r = -0.037 p = 0.889	**r = 0.635 p = 0.006**	**r = 0.633 p = 0.006**	r = -0.217 p = 0.403	r = 0.070 p = 0.791	r = 0.079 p = 0.762	r = -0.199 p = 0.443	r = -0.160 p = 0.514
Right Precuneus	18	-70	52	Neutral vs Emotional	r = 0.393 p = 0.119	r = -0.040 p = 0.880	r = 0.018 p = 0.945	r = 0.313 p = 0.221	r = 0.321 p = 0.209	r = 0.023 p = 0.929	r = -0.324 p = 0.204	r = -0.280 p = 0.276	r = -0.224 p = 0.387	r = 0.129 p = 0.623

Significant correlations (uncorrected for multiple comparisons) are emphasized in bold type Abreviations: dlPFC = dorsolateral Prefrontal Cortex.

Table 4

Significant correlations, between maternal sensitivity and BOLD activations when mothers see scenes of menacing vs prosocial adult male-female interactions, corrected for IPV-PTSD symptom severity.

Cluster Size	Peak Voxel t	Peak Voxel p	MNI Coordinates			Region	Overall Correlation	r within IPV-PTSD	p within IPV-PTSD	r within HC	p within HC	z-Score of the Difference Between the Corrected r of IPV-PTSD vs HC	Corrected r Between Maternal Sensitivity and Menacing vs Neutral Scenes	Corrected r Between Maternal Sensitivity and Prosocial vs Neutral Scenes	Mean Value (SD) Menacing vs Neutral Scenes	Mean Value (SD) Prosocial vs Neutral Scenes
			x	y	z		Corrected for IPV-PTSD Symptoms					Significance (p)				
**Positive Associations of BOLD Activity With Maternal Sensitivity**																
92	3.49	0.001	-9	-1	28	Mid Cingulate	0.308<br>0.471	0.310<br>0.324	0.227<br>0.221	0.483<br>0.501	0.027<br>0.024	-0.582<br>0.560	0.170<br>0.270	-0.358<br>0.017	-0.033<br>(0.15)<br>0.155	0.042<br>(0.19)<br>0.140
	3.26	0.001	-12	-13	31	Mid Cingulate										
	3.20	0.001	9	-13	28	Mid Cingulate										
27	3.60	<.001	-48	17	1	Left Inferior Frontal Gyrus	0.279<br>0.461	0.112<br>0.141	0.668<br>0.603	0.534<br>0.551	0.013<br>0.012	1.297<br>0.194	0.191<br>0.215	-0.214<br>0.163	0.076<br>(0.23)<br>0.029	-0.030<br>(0.24)<br>0.407
31	3.31	0.001	-15	-7	52	Medial Frontal Gyrus	0.348<br>0.467	0.366<br>0.368	0.014<br>0.161	0.476<br>0.447	0.029<br>0.048	0.257<br>0.797	-0.063<br>0.686	-0.431<br>0.004	0.003<br>(0.11)<br>0.864	0.019<br>(0.13)<br>0.338

	3.19	0.001	-15	-1	43	Mid Cingulate										
**Negative Associations of BOLD Activity With Maternal Sensitivity**																
411	4.09	<.001	-6	29	10	Ventral Anterior Cingulate										
	3.90	<.001	9	50	13	Ventromedial Prefrontal Cortex	-0.553<br>-0.557	-0.615<br>-0.613	0.009<br>0.012	-0.518<br>-0.536	0.016<br>0.015	0.312<br>0.754	-0.262<br>0.086	0.415<br>0.005	-0.052<br>(0.14)<br>0.016	-0.043<br>(0.20)<br>0.146
	3.84	<.001	-6	44	-17	Medial Orbitofrontal Cortex										
41	4.15	<.001	54	-22	-20	Right Inferior Temporal Gyrus	-0.544<br>-0.508	-0.603<br>-0.639	0.010<br>0.008	-0.437<br>-0.495	0.048<br>0.027	0.580<br>0.561	-0.523<br><.001	0.137<br>0.375	0.149<br>(0.18)<br><.001	0.039<br>(0.16)<br>0.113

Table 5

Significant correlations, between maternal sensitivity and BOLD activations when mothers see scenes of emotional (meancing and prosocial combined) vs neutral adult male-female interactions, corrected for IPV-PTSD symptom severity.

Cluster Size	Peak Voxel t	Peak Voxel p	MNI Coordinates			Region	Overall Correlation	r within IPV-PTSD	p within IPV-PTSD	r within HC	p within HC	z-Score of the Difference Between the Corrected r of IPV-PTSD vs HC	Corrected r Between Maternal Sensitivity and Menacing vs Neutral Scenes	Corrected r Between Maternal Sensitivity and Prosocial vs Neutral Scenes	Mean Value (SD) Menacing vs Neutral Scenes	Mean Value (SD) Prosocial vs Neutral Scenes
			x	y	z		Corrected for IPV-PTSD Symptoms					Significance (p)				
**Negative Associations of BOLD Activity With Maternal Sensitivity**																
496	4.23	<.001	42	2	28	Right Precentral Gyrus	-0.436<br>-0.556	-0.313<br>-0.339	0.222<br>0.199	-0.636<br>-0.620	0.002<br>0.004	1.010<br>0.313	-0.501<br>0.001	-0.520<br><.001	0.086<br>(0.13)<br><.001	0.025<br>(0.10)<br>0.122
	3.87	<.001	18	5	37	Mid Cingulate										
	3.78	<.001	30	5	40	Right dlPFC										
32	3.29	0.001	-45	8	25	Left dlPFC	-0.340<br>-0.421	-0.134<br>-0.164	0.608<br>0.543	-0.585<br>-0.566	0.005<br>0.009	1.292<br>0.196	-0.421<br>0.004	-0.395<br>0.008	0.133<br>(0.19)<br><.001	0.012<br>(0.16)<br>0.604
	3.14	0.002	-51	11	19	Left dlPFC										
27	3.19	0.001	-27	5	58	Left dlPFC	-0.387<br>-0.437	-0.163<br>-0.160	0.533<br>0.544	-0.518<br>-0.491	0.016<br>0.028	1.020<br>0.307	-0.429<br>0.004	-0.313<br>0.038	0.172<br>(0.19)<br><.001	0.008<br>(0.18)<br>0.769
41	3.25	0.001	-39	-10	43	Left Precentral Gyrus	-0.321<br>-0.464	-0.295<br>-0.319	0.250<br>0.229	-0.582<br>-0.560	0.006<br>0.010	0.820<br>0.412	-0.360<br>0.017	-0.470<br>0.001	0.070<br>(0.14)<br>0.002	0.022<br>(0.13)<br>0.283
	3.13	0.002	-15	-13	40	Mid Cingulate										
	2.96	0.003	-30	-4	46	Left										

						Precentral Gyrus										
35	3.17	0.001	18	-70	52	Right Precuneus	-0.293	0.010	0.969	-0.564	0.008	1.582	-0.445	-0.371	0.476 (0.30)	0.129 (0.27)
	3.08	0.002	15	-82	46	Right Precuneus	-0.463	-0.021	0.938	-0.540	0.014	0.113	0.002	0.013	<.001	0.002

Abreviations: dlPFC = dorsolateral Prefrontal Cortex.
